# *QuickStats*: Age-Adjusted Death Rates* for Drug Overdose,^^†^^ by Race/Ethnicity — National Vital Statistics System, United States, 2015–2016

**DOI:** 10.15585/mmwr.mm6712a9

**Published:** 2018-03-30

**Figure:**
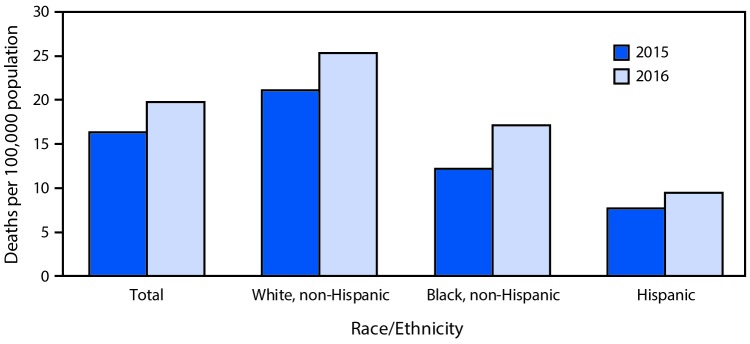
During 2015–2016, the age-adjusted death rates from drug overdose for the total population increased from 16.3 per 100,000 standard population to 19.8 (21.5%). The rate increased from 21.1 to 25.3 (19.9%) for non-Hispanic whites, from 12.2 to 17.1 (40.2%) for non-Hispanic blacks, and from 7.7 to 9.5 (23.4%) for Hispanics.

**Source:** National Vital Statistics System, Underlying cause of death data, 1999–2016. https://wonder.cdc.gov/ucd-icd10.html.

For more information on this topic, CDC recommends the following link: https://www.cdc.gov/drugoverdose/.

